# Long-Term Protective Immunity against *Ehrlichia chaffeensis* Infection Induced by a Genetically Modified Live Vaccine

**DOI:** 10.3390/vaccines12080903

**Published:** 2024-08-09

**Authors:** Swetha Madesh, Jodi McGill, Deborah C. Jaworski, Jonathan Ferm, Huitao Liu, Shawna Fitzwater, Paidashe Hove, Dominica Ferm, Arathy Nair, Cheyenne A. Knox, Kimia Alizadeh, Ashley Thackrah, Roman R. Ganta

**Affiliations:** 1 Center of Excellence for Vector-Borne Diseases, Department of Diagnostic Medicine/Pathobiology, College of Veterinary Medicine, Kansas State University, Manhattan, KS 66506, USA; smadesh@vols.utk.edu (S.M.); deborcj@gmail.com (D.C.J.); jdf7hw@missouri.edu (J.F.); hlnmb@missouri.edu (H.L.); sfitzwater@ku.edu (S.F.); paidah2000@gmail.com (P.H.); dgm5m@missouri.edu (D.F.); arathynirazhi@gmail.com (A.N.); cheyenneaknox@gmail.com (C.A.K.); kimdvm@illinois.edu (K.A.); cricket1@vet.k-state.edu (A.T.); 2 Department of Veterinary Pathobiology, College of Veterinary Medicine, Bond Life Sciences Center, University of Missouri, Columbia, MO 65211, USA; 3 Department of Veterinary Microbiology & Preventive Medicine, College of Veterinary Medicine, Iowa State University, Ames, IA 50011, USA; jlmcgill@iastate.edu

**Keywords:** tick-borne disease, monocytic ehrlichiosis, modified live vaccine, *Ehrlichia chaffeensis*, immune protection, long-lasting immunity, rickettsiales

## Abstract

Human monocytic ehrlichiosis, an emerging tick-borne disease, is caused by *Ehrlichia chaffeensis*. Infections with the pathogen are also common in the canine host. Our previous studies demonstrated that functional disruption within the *E. chaffeensis* phage head-to-tail connector protein gene results in bacterial attenuation, creating a modified live attenuated vaccine (MLAV). The MLAV confers protective immunity against intravenous and tick transmission challenges one month following vaccination. In this study, we evaluated the duration of MLAV protection. Dogs vaccinated with the MLAV were challenged with wild-type *E. chaffeensis* via intravenous infection at 4-, 8-, and 12-months post-vaccination. Immunized dogs rapidly cleared the wild-type pathogen infection and tested positive for bacteremia less frequently than unvaccinated controls. While immune responses varied among dogs, vaccinees consistently mounted IgG and CD4+ T-cell responses specific to *E. chaffeensis* throughout the assessment period. Our findings demonstrate that MLAV-mediated immune protection persists for at least one year against wild-type bacterial infection, marking a major advancement in combating this serious tick-borne disease. The data presented here serve as the foundation for further studies, elucidating the molecular mechanisms underlying virulence and vaccine development and aiding in preventing the diseases caused by *E. chaffeensis* and other tick-borne rickettsial pathogens.

## 1. Introduction

Human monocytic ehrlichiosis (HME) is caused by *Ehrlichia chaffeensis*, an *Amblyomma americanum* tick-transmitted pathogen [1,2,3]. While white-tailed deer are regarded as the reservoir of infection, people and dogs are incidental hosts in acquiring pathogen infections [4,5,6,7]. HME can cause significant morbidity in the majority of cases and can also result in fatalities in elderly and immune-compromised people and children [3,8,9,10]. The pathogen can also persist in people who have recovered from infection and, thus, pose a high risk to patients receiving blood transfusions and organ transplants from donors who previously tested positive for HME [11,12]. Doxycycline is the primary treatment option for HME, and a vaccine to prevent the disease is currently not available [13,14]. The canine host and *A. americanum* ticks are ideal for performing in vivo experiments, including assessing vaccine candidates, as they acquire infections naturally [15,16]. 

Developing vaccines against HME and other related rickettsial diseases remains a high priority, considering the continued emergence and rise in annual reported cases. Only limited work is described in the literature on vaccine research that is primarily focused on subunit vaccines. Transient immune protection against *E. chaffeensis* infection in the murine host (a host not known to acquire the infection) is documented when animals are immunized with a p28 outer membrane protein (p28-Omp) [17,18]. The murine host model is also used to report partial protection against *Ehrlichia ruminantium* when animals are vaccinated with a DNA vaccine targeted to a p28-Omp homolog [19]. Attenuated bacterial vaccines have also been assessed as live vaccines against *Ehrlichia ruminantium* in sheep and goats and *E. canis* in dogs [20,21]. Our recent studies demonstrated that several insertion mutations in the *E. chaffeensis* genome cause bacterial growth attenuation in dogs [22]. Infection with a mutation in the *E. chaffeensis* phage head-to-tail connector protein (*phtcp*) gene results in the pathogen’s attenuated growth in dogs and induces immunity to confer protection against infection challenge by intravenous (IV) infection or by tick transmission infection four weeks following the mutant’s inoculation [23,24]. The *phtcp* gene disruption mutation in *E*. *chaffeensis* limits bacterial zinc and iron acquisition [25]. We reported earlier that the gene deletion mutation in the *phtcp* ortholog of *Anaplasma marginale*, another tick-borne rickettsial pathogen, similarly results in the pathogen’s attenuated growth and induces protective immunity against virulent pathogen infection challenge [26].

In the current study, we report data demonstrating that the *E. chaffeensis phtcp* mutant as a live vaccine induces sufficient immune memory to offer protection for as long as one year against wild-type pathogen infections resulting from the IV injection challenge.

## 2. Materials and Methods

### 2.1. In Vitro Cultivation of E. chaffeensis

Wildtype and the *phtcp* mutant *E. chaffeensis* Arkansas isolate were cultivated in the canine macrophage cell line (DH82) (ATCC# CRL-10389) and in the ISE6 tick cell line (ATCC# CRL-3576) by following the methods previously described [27,28]. Briefly, a laboratory stock of wildtype and the *phtcp* mutant of *E. chaffeensis* Arkansas isolate stored in liquid nitrogen was thawed by warming to 37 °C in a water bath. The contents were then transferred to a tube with 10 mL of complete minimal essential media (MEM) and centrifuged at 4 °C for 10 min at 200× *g*. The culture pellet was resuspended in 1 mL MEM and transferred to a 25 cm^2^ tissue culture flask containing ~60% confluent monolayer of DH82 cells. The flask was then transferred to a 37 °C humidified incubator maintained with 5% CO_2_. The *E. chaffeensis* in ISE6 was similarly cultured, except that the L15B300 medium was used for cultures that were maintained at 34 °C in the absence of supplemented CO_2_. The infectivity in DH82 and ISE6 cultures was checked twice weekly by preparing cytospin slides, staining with polychromatic staining, and evaluating under a light microscope. The *phtcp* mutant *E. chaffeensis* cultures were used for vaccine inoculum preparations, and the wildtype *E. chaffeensis* cultures were used for infection studies when the infectivity reached about 80–90%. 

### 2.2. Animal Infections

Animal studies using the canine host were performed after receiving approval from the Institutional Animal Care and Use Committees (IACUC) of Kansas State University (KSU) (protocol approval # 4401) and according to the Public Health Service (PHS) policy on the humane care and use of laboratory animals and the U.S. Department of Agriculture’s Animal Welfare Act and Animal Welfare Regulations [29]. We purchased purpose-bred laboratory-reared beagle dogs from a USDA-approved vendor (Ridglan Farms, Inc., Mt. Horeb, WI, USA). Dogs of the approximate age of 8 months of either sex were injected with the *phtcp* mutant *E. chaffeensis* as the modified live attenuated vaccine (MLAV) as we previously performed [23]. The study included four groups of dogs; groups 1–3 represented vaccinations performed 4, 8, and 12 months prior to infection challenges, respectively, while group 4 dogs served as unvaccinated infection controls (Table 1). All three vaccination groups included three males and three females to assess if sex variation could contribute to vaccine protection. Group 4 had only three dogs (two males and a female) as similar infection experiments were performed earlier [23]. Challenge infections were performed after 4 months, 8 months, and 12 months following the MLAV infections with 1 mL each of wild-type *E. chaffeensis* cultures. Infections in dogs with the *phtcp* mutant or wild-type *E. chaffeensis* were performed as previously reported [23]. *E. chaffeensis* organisms were recovered from DH82 cultures when about 80% of cells were infected. The infected DH82 cells were pelleted by centrifugation at 200× *g*, media were discarded, and cells were resuspended in 1× phosphate-buffered saline (PBS) solution and concentrated again by 200× *g* centrifugation step. The washing step was repeated one additional time, and the final recovered cell pellets were resuspended to yield inoculum containing 2 × 10^8^ *Ehrlichia* organisms per ml in PBS. This inoculum was subsequently administered intravenously at a dosage of 1 mL per dog. To carry out infection challenges at the same time, MLAV vaccinations were performed at different times for the dogs in groups 1–3; the vaccinations were performed 4, 8, and 12 months prior to challenge day, and the inoculum used for the wild-type bacterial infection was prepared from the same batch of culture.

### 2.3. E. chaffeensis-Specific PCR

Four milliliters of blood were collected in EDTA tubes on day zero before vaccination and at various time points after MLAV or infection challenges (harvest dates for each group varied slightly and were identified in the Section 3). Plasma and buffy coats were obtained from processing the blood samples and used for assessing infection status by nested PCR analysis and ELISA for antibody analysis, as previously described [15,23]. DNAs from blood samples were extracted using the Qiagen Blood and Tissue Kit (Qiagen, Germany) and used for evaluating mutant infection status by performing nested PCR targeting the insertion segment region-specific primers (primers: 1st PCR; forward, 5′GGCCATCTCTCCCCTTAACA; reverse, 5′TATCCCTTATGTTACGATAACTTA; annealing temperature, 54 °C and 2nd PCR primers: forward, 5′TGTACCTGTATCCTCACCTATCACC; reverse, 5′TGCAACAGTTATTTAATGTATGGTTG; annealing temperature, 57 °C). Similarly, wild-type *E. chaffeensis* infection status was assessed using nested PCR targeting the p28-Omp 14 gene (Ech_1136) as we previously described in [23]. Briefly, two microliters of purified genomic DNA from dog blood were used in the first-round PCRs in a 25 µL reaction volume with Go Taq Green DNA polymerase per the manufacturer’s instructions (Promega, Madison, WI, USA). For the second-round PCRs, 2 µL of 1:100 diluted first-round PCR products were used as templates using the nested PCR primer sets. The amplicons from 2nd PCR were analyzed on a 1.5% ethidium bromide-stained agarose gel to identify target PCR products [30].

### 2.4. ELISA

*E. chaffeensis* total protein lysates were prepared from purified organisms isolated from DH82 and infected ISE6 cell cultures [15]. The protein concentration was determined and utilized for performing the enzyme-linked immunosorbent assay (ELISA) [15]. Plasma samples collected from dogs before infection and at various days post-infection were tested by ELISA for the presence of the *E. chaffeensis*-specific total IgG using the 1:200 diluted samples, with all assays performed in triplicate wells [15].

### 2.5. Preparation and Peripheral Blood Mononuclear Cells (PBMCs)

About 8–10 mL volume of blood samples were collected from each animal in sodium citrate CPT tubes (BD Biosciences, San Jose, CA, USA) at the indicated time points after MLAV vaccination and following IV infection of wild-type *E. chaffeensis* [24]. The blood tubes were centrifuged as per the manufacturer’s instructions prior to shipping overnight to Iowa State University in cold packs. The harvested PBMCs were washed twice with cRPMI media; cells were counted and adjusted to 4 × 10^6^ PBMCs/mL. 

### 2.6. ELISPOT Assays

ELISPOT assays were performed using the ELISpot PLUS Canine IFN-γ (ALP) kit (Mabtech, Inc., Cincinnati, OH, USA). Pre-coated ELISPOT plates were washed once with PBS, followed by performing blocking for 1 h with cRPMI. Subsequently, samples were plated at 100 μL of cells/well in duplicate wells. Cells were stimulated with 10 μg/mL of purified *E*. *chaffeensis* lysate prepared from DH82 cell cultures. In control wells, PBMCs were maintained only in cRPMI media with no antigen added. To serve as a positive control, PBMCs were stimulated with 5 μg/mL Concanavalin A (Sigma-Aldrich, St. Louis, MO, USA). The number of IFN-γ spot forming units in the mock control was subtracted to account only for the *E. chaffeensis*-specific response. The culture plates were wrapped in foil and incubated overnight at 37 °C, then washed and developed as per the manufacturer’s instructions to measure IFN-γ expression. After developing, the plates were allowed to air dry and subsequently stored in a cool, dark location until assessed using a CTL Immunospot S5 Fluorcore ELISpot analyzer. 

### 2.7. ELISA for IFNγ

PBMCs were also prepared for ELISA assays in parallel with ELISpot assays. PBMCs were plated in duplicate wells with cRPMI and stimulated using *E. chaffeensis* whole-cell antigens or Concanavalin A or having no antigen added, as indicated in the previous section [24]. The cells were then incubated for 72 h in a 37 °C humidified incubator. Cell culture supernatants recovered from duplicate wells were pooled and stored at −70 °C until analysis. The IFNγ concentrations in the supernatants were assessed using a commercial ELISA kit (R&D Systems, Minneapolis, MN, USA) as per the manufacturer’s instructions.

### 2.8. Intracellular Cytokine Staining and Flow Cytometry

Intracellular cytokine analysis was conducted by flow cytometry, as we previously described [24]. Briefly, PBMCs were cultured and stimulated with the addition of Brefeldin A during the final 5–6 h of incubation. Following stimulation, cells were surface stained with mouse anti-canine CD3-FITC (clone CA17.2112), CD4- PECy7 (clone YKIX302.9), rat anti-dog CD44-AlexaFluor 488 (clone YKIX337.8.7), and mouse anti-human CD62L-APC-Cy7 (clone FMC46) mouse-anti-bovine IFNγ-APC (clone CC302), obtained from BioRad Laboratories (Hercules, CA, USA). Subsequently, PBMCs were resuspended in FACS buffer (0.1% NaN_3_, 10% fetal calf serum, PBS) and incubated with primary antibodies at 10 μg/mL for 20 min at 4 °C. Cells were then fixed using BD FACS Lysis buffer (BD Biosciences, San Jose, CA, USA). Cytokine intracellular staining for IFNγ was performed using the BD Fixation and Permeabilization Solution kit (BD Biosciences, San Jose, CA, USA). Flow cytometry data were acquired on a BD FACSCanto flow cytometer and analyzed with FlowJo software Version 10.10 (BD Biosciences, San Jose, CA, USA), employing a gating strategy as illustrated in Supplemental Figure S1.

### 2.9. Statistical Analysis

Statistical analyses were conducted to examine differences in the positive/negative infection status over time in dogs infected with wild-type *E. chaffeensis* and MLAV. Statistical analysis in GraphPad Prism 10 was performed to analyze differences in the percent positive PCR results between MLAV vaccinate and wild-type control infected dogs by one-way ANOVA with multiple comparisons. A binomial generalized estimating equation model was fit using Stata 12 software (StataCorp LP, College Station, TX, USA) to account for repeated measures on dogs over time using a logit link and exchangeable correlation. Additionally, differences in ELISA on the final sampling day between dogs infected with MLAV and those infected with wild-type *E. chaffeensis* were evaluated using a 2-tailed unpaired Student’s *t* test (GraphPad Software, La Jolla, CA, USA).

## 3. Results

### 3.1. E. chaffeensis MLAV Vaccination

In the present study, the length of protection offered by the MLAV against IV infection challenge was assessed in three groups of dogs following 4-, 8-, and 12-months post *phtcp* mutant injections (groups 1, 2, and 3), respectively, and group 4 dogs served as unvaccinated infection controls (Table 1). 

**Figure 1:**
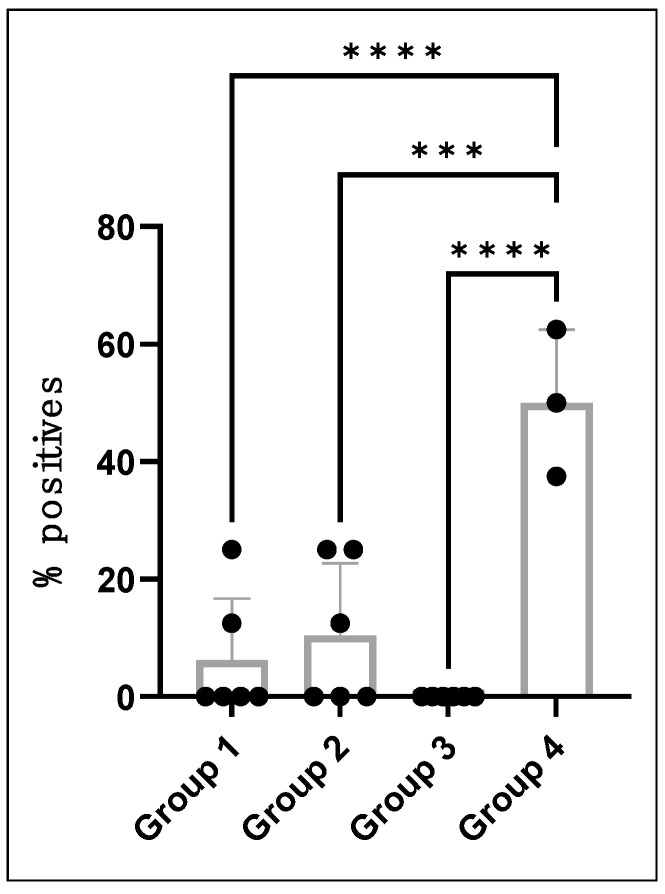
MLAV vaccination induced a significant drop in systemic infection. We assessed the differences in the percent positives of PCR detection of infection in the three vaccinated groups and compared them with unvaccinated groups for statistical significance. The one-way ANOVA with multiple comparisons identified significant differences between Groups 1, 2, and 3 compared to Group 4 (unvaccinated) with *p* < 0.0001 (****) for Groups 1 and 3 and *p* = 0.0001 (***) for Group 2. There was no significant difference observed when we compared among the three vaccinated groups: Groups 1, 2, and 3.

MLAV vaccination reduced systemic *E. chaffeensis* infection following intravenous challenge after 4-, 8- and 12-months post-vaccination. Blood samples from all three vaccinated groups tested negative after vaccination when assessed using nested PCR targeting the MLAV-specific DNA segment (Table 2). Following the infection challenge, MLAV-vaccinated dogs tested positive less frequently (Table 3) compared to dogs in the unvaccinated groups (Table 4), independent of 4-, 8-, or 12-month post-vaccination status. Only two of six dogs in the 4-month post-vaccination group (group 1) were positive for *E. chaffeensis* and for three time points: dog # 43-21 on day 7 and dog # 45-21 on days 10 and 14 post-infection challenge tested positive. In the 8-month post-vaccination group (group 2), three of the six dogs tested positive at five time points: dog # 01-21 on days 14 and 18, dog # 03-21 on day 7, and dog # 07-21 on days 14 and 22 tested PCR positive. All six dogs in group 3 tested negative for infection after the infection challenge. All dogs in the unvaccinated group tested positive more frequently throughout the assessment period (Table 4). In total, dogs in the three vaccination groups tested positive for the *E. chaffeensis* infection only 5.5% of the time, whereas the unvaccinated dogs following the IV infection challenge tested positive 50% of the time (Table 5). The overall vaccine protection efficiency calculated for the detection of *E. chaffeensis* positives in the MLAV-vaccinated dogs compared to unvaccinated wild-type infected dogs was 89% [31]. Statistical analysis performed by a one-way ANOVA or the Welch’s *t*-test revealed no significant differences in vaccine protection among the three vaccinated groups, whereas significant differences were observed when comparing the dogs testing positive for the systemic bacterial presence in vaccinated groups with the unvaccinated group dogs (Figure 1).

### 3.2. Antibody Responses Observed in MLAV-Vaccinated Dogs after Vaccination and Following Infection Challenge

**Figure 2:**
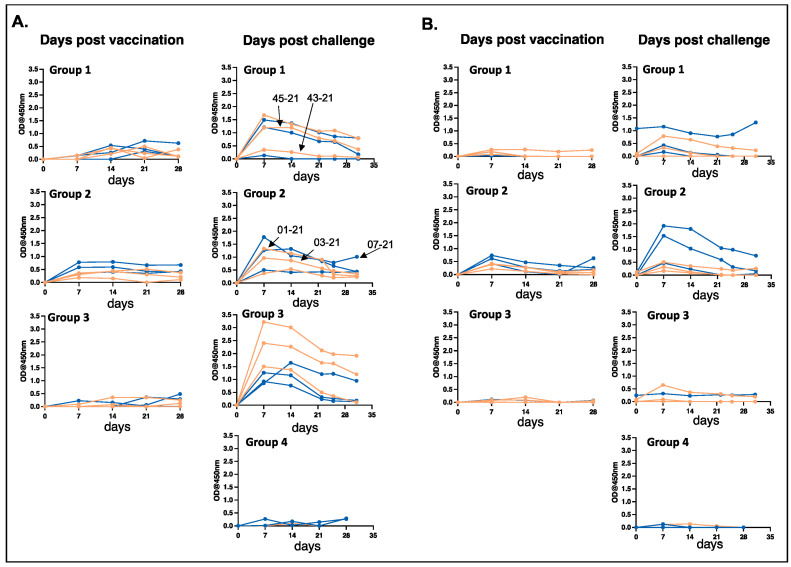
Antibody response of dogs after MLAV vaccination and after wild-type *E. chaffeensis* infection challenge. *E. chaffeensis*-specific IgG was measured in plasma at the indicated time points following vaccination and wild-type challenge for dogs immunized 4, 8, and 12 months prior to infection (groups 1, 2, and 3, respectively). Group 4 dogs were unvaccinated and served as infection controls. IgG responses were analyzed by coating ELISA plates with *E. chaffeensis* antigens generated from cultures that were either grown in DH82 canine macrophage cell line ((**A**) left panels) or ISE6 tick cells ((**B**) right panels). The lines represent the dogs in each group. Females and males were identified with pink and blue lines, respectively. Dogs positive for *E. chaffeensis* bacteremia at least once following the infection challenge were indicated by arrows in the post-infection challenge images in panel (**A**).

We measured *E. chaffeensis*-specific IgG responses in all dogs in the three vaccinated groups following vaccination and after infection challenge. Similarly, the IgG response in unvaccinated dogs following infection was assessed (Figure 2). The IgG response was observed for *E. chaffeensis* antigens generated from cultured organisms recovered from the macrophage cell line (DH82) as well as the antigens prepared from ISE6 tick cell cultured bacteria (Figure 2). All vaccinated animals generated an IgG response, which was enhanced after the infection challenge. Some dogs had greater antibody response compared to others, particularly following the infection challenge. The IgG response was similar for the bacterial antigens prepared from the organisms cultured in the macrophage cell line or in the tick cell line, except for the group 3 dogs (Figure 2).

### 3.3. Vaccination and Wild-Type E. chaffeensis Challenge Induce Antigen-Specific Cellular Immune Responses

**Figure 3:**
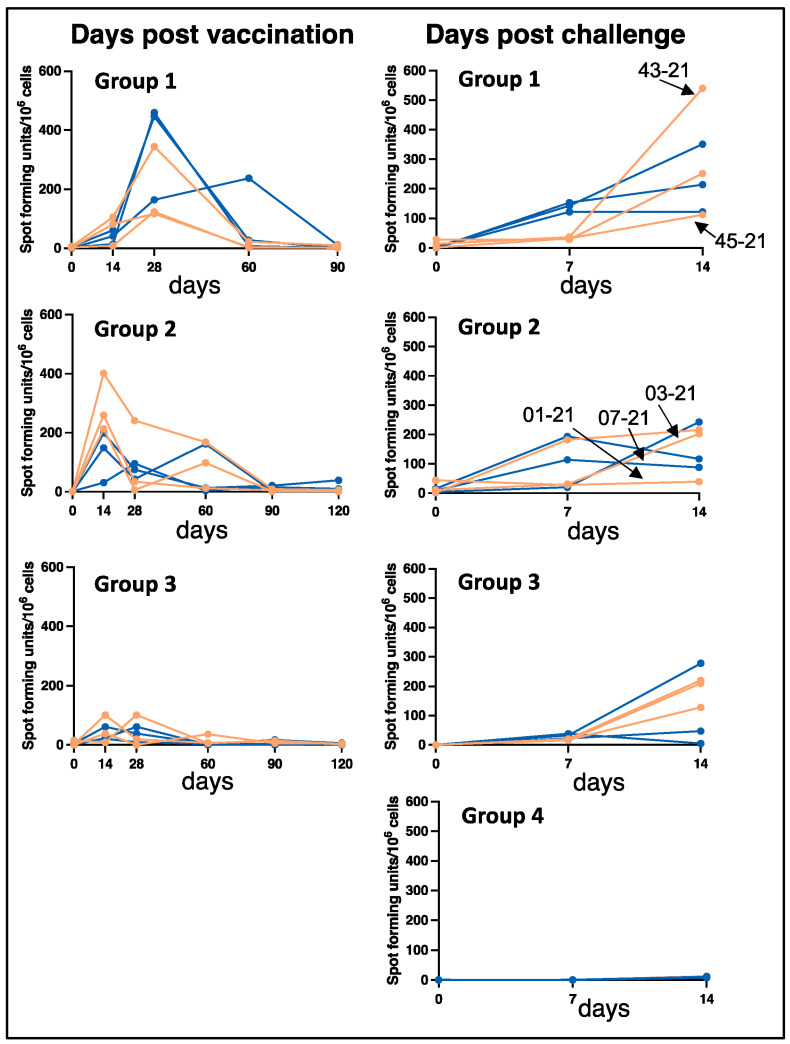
IFNγ ELISpot responses of dogs following MLAV vaccination and after wild-type infection challenge. PBMCs were isolated at the indicated times following vaccination for the three vaccinated groups ((**left**) panel) and after infection challenge for all four groups ((**right**) panel) and plated at a concentration of 4 × 10^5^ cells/well on prepared ELISPOT plates. Cells were stimulated in duplicate wells with 10 μg/mL *E. chaffeensis* host cell-free lysate. Positive control wells were stimulated with concanavalin A. Negative control wells were stimulated with media only. After overnight culture, ELISPOT plates were developed, and spot-forming units were enumerated using an ELISPOT reader. The lines represent the dogs in each group. Females and males were identified with pink and blue lines, respectively. Dogs positive for *E. chaffeensis* bacteremia at least once following the infection challenge were indicated by arrows.

**Figure 4:**
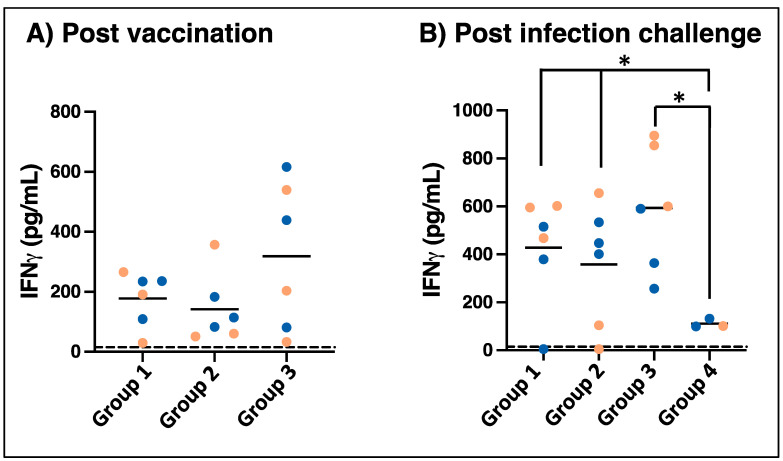
IFNγ ELISA responses of dogs following MLAV vaccination (panel (**A**)) and 4, 8, or 12 months later by intravenous challenge with wild-type *E. chaffeensis* (panel (**B**)). PBMCs were isolated and plated, as in Figure 3. Cells were stimulated, as shown in Figure 3, except that the cultures were maintained for 72 h. Culture supernatants were analyzed by commercial sandwich ELISA for IFNγ [*p* = 0.029 (*) for vaccinated compared to unvaccinated dogs] (Pink and blue bullets refer to female and male dogs in the study, respectively).

*E. chaffeensis*-specific cellular immune responses were measured by IFN-γ ELISPOT in peripheral blood from vaccinated animals at multiple intervals following vaccination in all three groups (Figure 3, left panel). *E. chaffeensis*-specific IFN-γ- producing cells measurable by ELISPOT were observed in most of the animals, with a peak response detected for samples from days 14–28 post-vaccination, although the kinetics varied widely among different dogs. Cellular immune responses were similarly assessed for the blood samples collected on days 7 and 14 post-infection challenges (Figure 3, right panel). Cellular IFN-γ responses were absent or slow to develop in unvaccinated infection-control animals. In contrast, cellular responses were not detectable on day zero prior to infection challenges in the vaccinated animals, but the number of IFN-γ spot-forming units increased rapidly and was detectable by 7 to 14 days post-challenge in 15 out of 18 immunized dogs. A total of three dogs had limited or absent IFN-γ ELISPOT responses following the challenge: one in group 2 (dog # 01-21) and two in group 3 (dog #s 09-20 and 11-20). In parallel with ELISPOT assays, *Ehrlichia*-specific IFN-γ secretion was observed in the PBMCs stimulated with macrophage culture-derived *E. chaffeensis* when assessed after one month post-vaccination for all groups (Figure 4A) and day 14 post-challenge (Figure 4B). Consistent with the ELISPOT results, most dogs exhibited *E. chaffeensis*-specific IFN-γ responses by ELISA that were detected one month post-challenge. Following wild-type pathogen infection, IFN-γ concentration increased in dogs from groups 1 and 3 compared to the unvaccinated controls in group 4 (Figure 4B).

**Figure 5:**
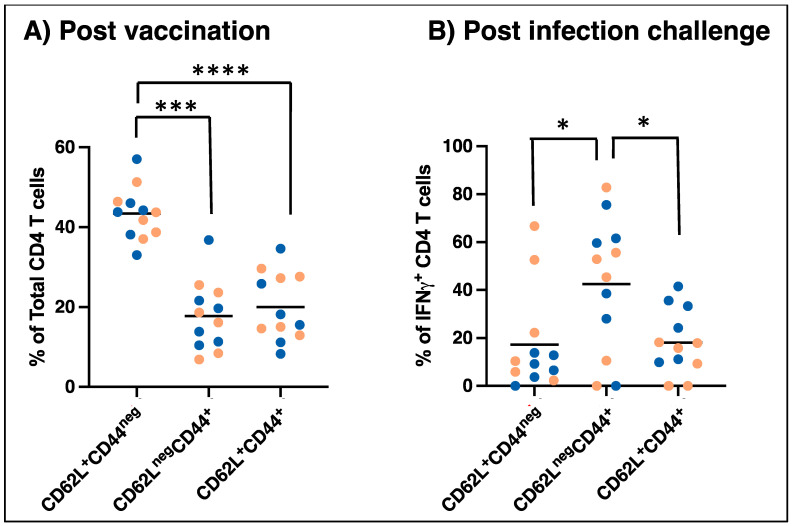
Memory CD4 T cell populations in dogs following MLAV vaccination (**A**) and followed 4, 8, or 12 months later by wild-type *E. chaffeensis* IV challenge (**B**). PBMCs were isolated from vaccinated dogs at 8 weeks post-vaccination and stimulated overnight in duplicate wells with 10 μg/mL *E. chaffeensis* host cell-free lysate, and the assays included positive and controls, as in Figure 3. In the final 5–6 h of culture, Brefeldin A was added to each culture. Cells were then surface stained with antibodies specific to canine CD3, CD4, CD44, and CD62L, and stained intracellularly for IFNγ and used for flow cytometry analysis. Total and *E. chaffeensis*-specific (gated on IFNγ+) CD4 T cells were analyzed to identify naïve (CD62L^+^CD44^neg^), effector memory T cells (CD62L^neg^CD44^+^), and central memory T cells (CD62L^+^CD44^+^). The dots represent the dogs in each group. Females and males were identified with pink and blue dots, respectively [*p* = 0.0001 or <0.0001 (*** or ****) for differences between the three naïve, Tcm, and Tem subsets. *p* = 0.016 (*) for differences between the three IFNg+ naïve, Tcm, and Tem subsets] (Pink and blue bullets refer to female and male dogs in the study, respectively).

To determine if vaccination induced memory responses and the development of central memory T cells, PBMCs from infection groups following vaccination (groups 2, 3) were assessed for samples four months after vaccination. Total (Figure 5A) and antigen-specific (IFN-γ positive) (Figure 5B) CD4^+^ T cells were assessed by flow cytometry for expression of CD44 and CD62L to identify subsets of memory populations. CD4^+^ T cells were identified as naïve (CD62L^+^CD44^neg^), effector memory T cells (CD62L^neg^CD44^+^), and central memory T cells (CD62L^+^CD44^+^) using the gating strategy described by Rothe et al. [32]. As seen in Figure 5A, approximately half of all circulating cells were CD62L^+^CD44^neg^, defined as naïve CD4 T cells, with fewer circulating effector and central memory populations. In contrast, a much higher proportion of *E. chaffeensis*-specific T cells were CD62L^neg^CD44^+^, defined as effector memory T cells, with lower proportions defined as CD62L^+^CD44^neg^ (naïve) or CD62L^+^CD44^+^ (central memory).

## 4. Discussion

In the current study, we extend our *E. chaffeensis* MLAV vaccine study to demonstrate that the vaccine protection offered by the *phtcp* mutant is effective for up to one year. Vaccines against intracellular pathogens likely require activity from both the innate and adaptive arms of the immune system, which is achievable with modified live vaccines. Live vaccines are also expected to provide long-lasting immunity. Indeed, our study suggests that the *E. chaffeensis* MLAV offers long-lasting protection demonstrated up to one year. Consistent with these results, we have documented the induction of *E. chaffeensis*-specific B cell and T cell responses, including having an expanded memory T cell population following MLAV vaccination. We observed high variation in the IgG responses. While most dogs in groups 1 and 2 showed no major differences in the IgG against bacterial antigens originating from macrophage and tick cell cultures, group 3 dogs exhibited a higher IgG response to macrophage culture-derived antigens compared to tick cell-derived antigens. It is not clear why this difference is noted for this group; however, it had no impact on the MLAV immune protection. Notably, variations in IgG responses in dogs did not correlate well with immune protection, as all dogs in all vaccinated groups following the infection challenge had significantly fewer infection positives for all three vaccinated groups compared to unvaccinated groups. 

We observed the complete clearance of infection for dogs in the 12-month post-vaccination group, while occasional positives were detected in the 4-month and 8-month post-vaccination groups. Dogs used in the study represent outbred animals; thus, animal-to-animal variations are anticipated to cause an infection due to some genetic variation. Secondly, rickettsial infections are known to differ for different age groups, such as with *Rickettsia rickettsii* infection, which, in children, results in a more severe clinical disease [33]. The dogs in the 12-month vaccination group are older by 8 months and 4 months, respectively, compared to those in groups 1 and 2. Thus, better infection clearance in 12 months post-vaccination may possibly be the result of an age-specific response. This hypothesis, however, remains to be tested. Importantly, among the three vaccinated groups, there was no significant difference noted for the infection, whereas significant differences were observed for the vaccinated groups compared to the unvaccinated group. We observed cyclical changes in some animals testing positive, followed by negative and, again, positive for infection at later time points. The cyclical rickettsemia observed in the current study is similar to our several prior studies with *E. chaffeensis* [23,24]. Cyclical rickettsemia is well documented in a related rickettsial *Anaplasma marginale* [34] resulting from the immune evasion strategy involving antigenic variation [35]. Little is known about the molecular basis for *E. chaffeensis* for the observed cyclical rickettsemia. 

Prior studies using murine models using different *Ehrlichia* species pathogens reported that CD4^+^ T cell-mediated IFNγ production is essential for mitigating a fatal outcome from an infection [36,37,38]. We previously demonstrated that immunization with the live *E. chaffeensis phtcp* mutant as the MLAV produces Th1 CD4^+^ T cell immunity in a physiologically relevant canine infection model [23,24]. We also presented evidence that MLAV-vaccinated dogs develop protection from wild-type *E. chaffeensis* received via needle inoculation, as well as the infection challenge by tick transmission when protection was tested one month following vaccination. Also, using the canine model, Budacheria et al. (2022) reported that vaccination with a recombinant VirB2-4, an *E. chaffeensis* type IV secretion system protein, induces potent IFNγ responses as measured by ELISPOT and qRT-PCR analysis in the circulating PBMCs with conferring protection from tick-transmitted infection challenge. Interestingly, vaccination with either the OMP-1B protein or entry-triggering protein EtpE induces less IFNγ and is unable to protect dogs from tick-transmitted *E. chaffeensis* infection [39]. In the current study, we detected a peak of systemic *E. chaffeensis*-specific IFNγ+ T cells around 14 to 28 days after MLAV vaccination, although some dogs had a lower response. The number of IFNγ-producing T cells increased in most vaccinated dogs following the infection challenge. Of the 18 vaccinated dogs in the three groups, all but 3 had higher *E. chaffeensis*-responsive T cells following infection challenge. Of those, one dog was vaccinated 8 months prior to the infection challenge (group 2: # 01-21), and the remainder were vaccinated 12 months prior to receiving infections (group 3: #s 09-20 and 11-20). Notably, these animals maintained a negative status for *E. chaffeensis* in their systemic circulation. Thus, while the cellular immune response may appear diminished in some dogs 12 months following vaccination as per the parameters assessed in the current study, this did not correlate with the disease progression, suggesting that other factors contribute to the disease resistance in vaccinated dogs, such as the contributions from nonconventional lymphocyte populations and NK or NKT cells [36].

Vaccine-induced protection against intracellular bacterial infections, as reported for *Salmonella* species and *Mycobacterium* species, is attributed to the presence of central memory T cells [40,41,42,43]. Although effector memory CD4^+^ T cells are differentiated and composed of rapid effector functions, these cells are not known to persist for a long time. Central memory T cells are undifferentiated and take more time to activate but are highly proliferative and provide more potent recall responses. There is little known about the correlates of protection against *E. chaffeensis* beyond Th1 immunity; thus, it is not clear what role central and effector memory responses may play in controlling the infection. However, we observed that MLAV vaccination induced a population of long-lived CD4^+^ T cells. At 4 months post-vaccination, the T cells that produce IFNγ in response to in vitro stimulation were predominantly effector memory T cells, with a smaller population of central memory T cells found. This is not surprising, as this population would be expected to respond with more rapid cytokine production in the short-term intracellular cytokine assay. Additional work should focus on defining the importance of memory CD4^+^ T cells in protection from *E. chaffeensis* infection.

## 5. Conclusions

We demonstrate that MLAV vaccination induces *E. chaffeensis*-specific humoral and cellular immunity, which is likely important in offering protection for up to one year, possibly because of the presence of a long-lasting memory. The immune protection against wild-type *E. chaffeensis* was detected in a host naturally known to acquire infection for as long as a year post-vaccination. The current study also demonstrates that a functional defect in the *phtcp* gene in *E. chaffeensis* serves as an ideal live vaccine candidate for inducing long-lasting immunity.

## Figures and Tables

**Table 1:** Vaccination groups were used in the study.

Animal Groups	Females + Males	Challenge Time (Months Post Vaccination)
Group 1	3 + 3	4 months
Group 2	3 + 3	8 months
Group 3	3 + 3	12 months
Group 4	1 + 2	Unvaccinated

**Table 2:** Infection status over time post-MLAV vaccination *.

Group 1—4 Months Post-Vaccination								
**Animal #s**	**Day 0**	**Day 2**	**Day 7**	**Day 12**	**Day 14**	**Day 19**	**Day 22**	**Day 29**
43-21(F)	-	-	-	-	-	-	-	-
45-21(F)	-	-	-	-	-	-	-	-
47-21(F)	-	-	-	-	-	-	-	-
49-21(M)	-	-	-	-	-	-	-	-
50-21(M)	-	-	-	-	-	-	-	-
51-21(M)	-	-	-	-	-	-	-	-
**Group 2—8 Months Post-Vaccination**								
**Animal #s**	**Day 0**	**Day 3**	**Day 7**	**Day 10**	**Day 14**	**Day 17**	**Day 21**	**Day 28**
01-21(F)	-	-	-	-	-	-	-	-
02-21(F)	-	-	-	-	-	-	-	-
03-21(F)	-	-	-	-	-	-	-	-
07-21(M)	-	-	-	-	-	-	-	-
08-21(M)	-	-	-	-	-	-	-	-
09-21(M)	-	-	-	-	-	-	-	-
**Group 3—12 Months Post-Vaccination**								
**Animal #s**	**Day 0**	**Day 3**	**Day 7**	**Day 10**	**Day 14**	**Day 17**	**Day 22**	**Day 28**
01-20(F)	-	-	-	-	-	-	-	-
02-20(F)	-	-	-	-	-	-	-	-
06-20(F)	-	-	-	-	-	-	-	-
07-20(M)	-	-	-	-	-	-	-	-
09-20(M)	-	-	-	-	-	-	-	-
11-20(M)	-	-	-	-	-	-	-	-

* - or + refer to animals testing negative or positive for infection assessed by PCR, respectively.

**Table 3:** Infection status over time in vaccinated dogs in post-infection challenge *.

Group 1—4 Months Post-Vaccination Challenge								
**Animal #s**	**Day 3**	**Day 7**	**Day 10**	**Day 14**	**Day 18**	**Day 22**	**Day 25**	**Day 31/32**
43-21(F)	-	+	-	-	-	-	-	-
45-21(F)	-	-	+	+	-	-	-	-
47-21(F)	-	-	-	-	-	-	-	-
49-21(M)	-	-	-	-	-	-	-	-
50-21(M)	-	-	-	-	-	-	-	-
51-21(M)	-	-	-	-	-	-	-	-
**Group 2—8 Months Post-Vaccination Challenge**								
**Animal #s**	**Day 4**	**Day 7**	**Day 11**	**Day 14**	**Day 18**	**Day 22**	**Day 25**	**Day 31/32**
01-21(F)	-	-	-	+	+	-	-	-
02-21(F)	-	-	-	-	-	-	-	-
03-21(F)	-	+	-	-	-	-	-	-
07-21(M)	-	-	-	+	-	+	-	-
08-21(M)	-	-	-	-	-	-	-	-
09-21(M)	-	-	-	-	-	-	-	-
**Group 3—12 Months Post-Vaccination Challenge**								
**Animal #s**	**Day 4**	**Day 7**	**Day 11**	**Day 14**	**Day 18**	**Day 22**	**Day 25**	**Day 31/32**
01-20(F)	-	-	-	-	-	-	-	-
02-20(F)	-	-	-	-	-	-	-	-
06-20(F)	-	-	-	-	-	-	-	-
07-20(M)	-	-	-	-	-	-	-	-
09-20(M)	-	-	-	-	-	-	-	-
11-20(M)	-	-	-	-	-	-	-	-

* - or + refer to animals testing negative or positive for infection assessed by PCR, respectively.

**Table 4:** Infection status over time in unvaccinated dogs in group 4a after IV infection challenge *.

Animal #s	Day 4	Day 7	Day 11	Day 14	Day 18	Day 22	Day 24	Day 30
56-21(M)	+	+	+	+	+	-	-	-
57-21(M)	-	-	-	+	+	+	-	+
60-21(F)	-	+	+	+	-	-	-	-

* - or + refer to animals testing negative or positive for infection assessed by PCR, respectively.

**Table 5:** Combined data for all MLAV vaccinated and unvaccinated dogs post-infection challenges.

Vaccine/Infection	Group #s	Dogs Positive/Assessed	Frequency of Positives
Vaccinated and infected	1, 2, and 3	5/18 (28%)	8/144 (5.5%)
Unvaccinated and infected	4	3/3 (100%)	12/24 (50%)

## Data Availability

All data related to this manuscript are included in the manuscript.

## Supplementary Materials

The following supporting information can be downloaded at: https://www.mdpi.com/article/10.3390/vaccines12080903/s1, Figure S1: Representative gating strategy used to analyze memory T cell subsets from vaccinated dogs. Cells were strained using intracellular cytokine staining for IFN-gamma and then analyzed by flow cytometry. Cells were gated based on singlets, live/dead, and total CD4 T cells. *E. chaffeensis*-specific CD4 T cells were identified based on their production of IFN-gamma in response to WCS stimulation. Representative sample collected from a vaccinated dog 4 months post-vaccination.
