# Supplementary material for: Long-Term Protective Immunity against Ehrlichia chaffeensis Infection Induced by a Genetically Modified Live Vaccine
Source: Vaccines (Basel). 2024 Aug 9;12(8):903. doi: 10.3390/vaccines12080903 (PMC11360114; doi:10.3390/vaccines12080903)
Supplement: Supplementary file 1 [file vaccines-12-00903-s001.zip › vaccines-3088824-supplementary.pdf]

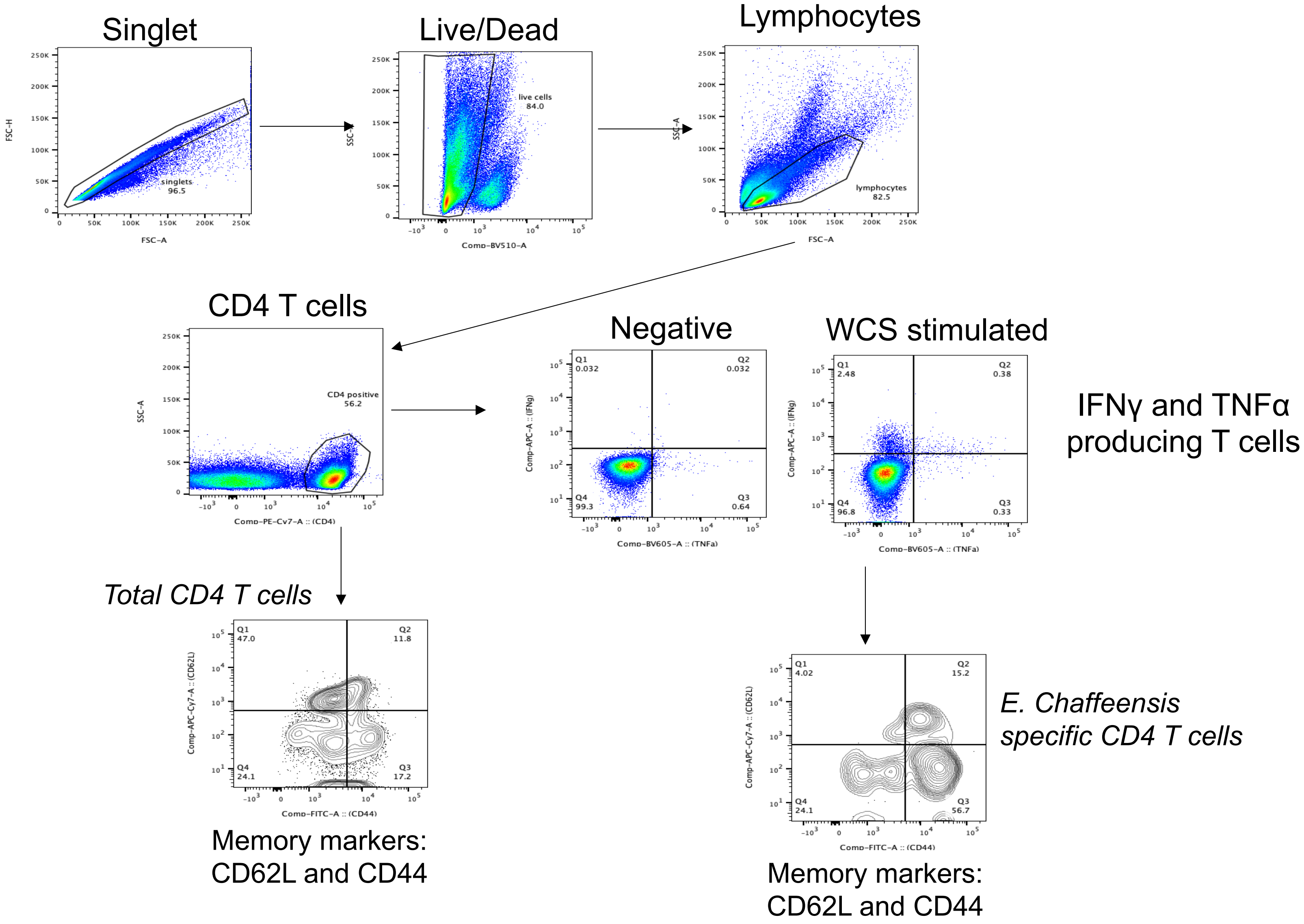

Figure S1. Representative gating strategy used to analyze memory T cell subsets from vaccinated dogs. Cells were strained using intracellular cytokine staining for IFN-gamma and then analyzed by flow cytometry. Cells were gated based on singlets, live/dead and total CD4 T cells. *E. chaffeensis* specific CD4 T cells were identified based on their production of IFN-gamma in response to WCS stimulation. Representative sample collected from a vaccinated dog at 4 months post vaccination.
